# Supplementary material for: Metabolic View on Human Healthspan: A Lipidome-Wide Association Study
Source: Metabolites. 2021 Apr 30;11(5):287. doi: 10.3390/metabo11050287 (PMC8146132; doi:10.3390/metabo11050287)
Supplement: Supplementary file 1 [file metabolites-11-00287-s001.zip › metabolites-1191027-supp-final/Fig_S1.pdf]

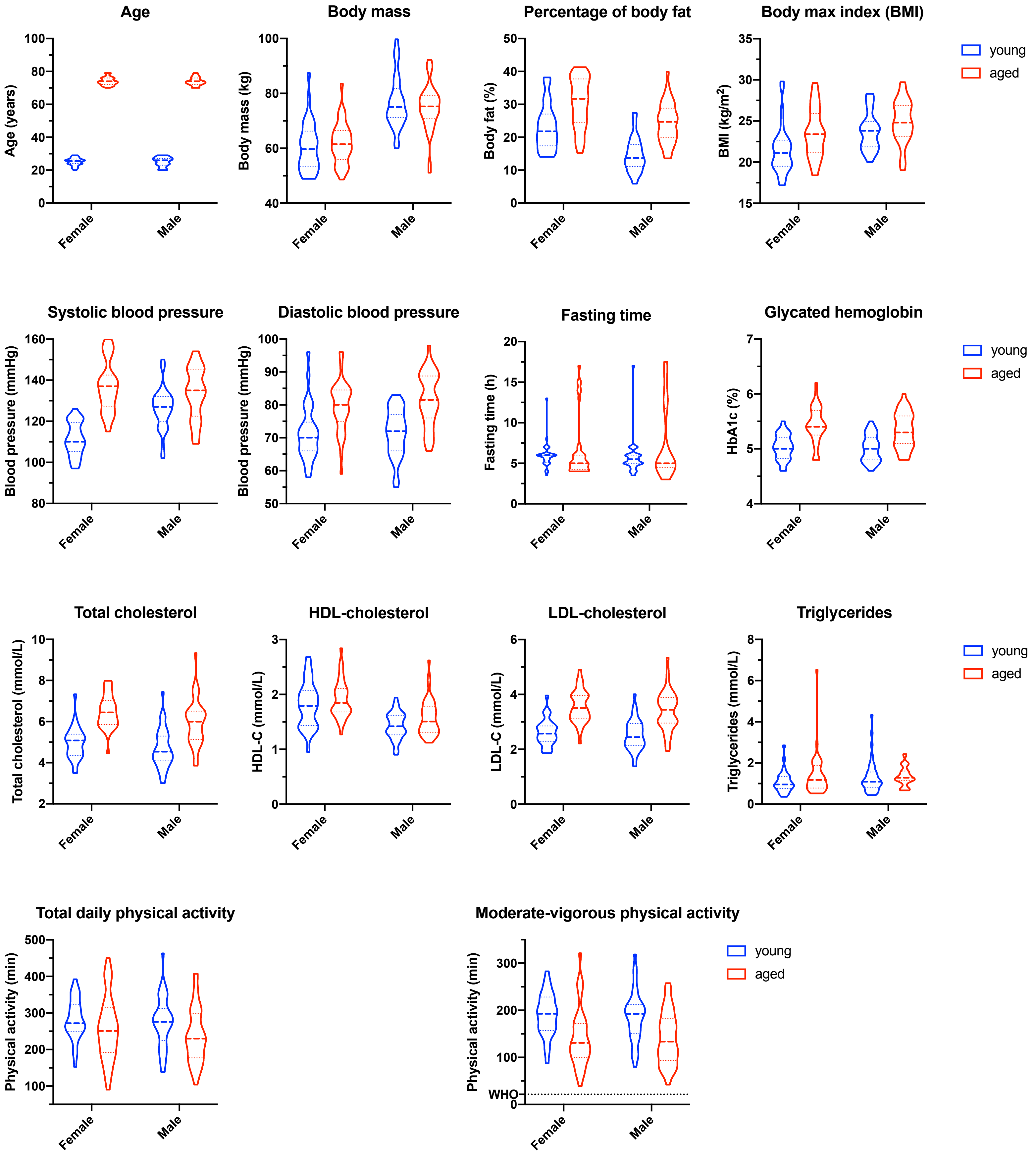
Age
Age (years)
Body mass
Body mass (kg)
Percentage of body fat
Body fat (%)
Body max index (BMI)
BMI (kg/m^2)
Systolic blood pressure
Blood pressure (mmHg)
Diastolic blood pressure
Blood pressure (mmHg)
Fasting time
Fasting time (h)
Glycated hemoglobin
HbA1c (%)
Total cholesterol
Total cholesterol (mmol/L)
HDL-cholesterol
HDL-C (mmol/L)
LDL-cholesterol
LDL-C (mmol/L)
Triglycerides
Triglycerides (mmol/L)
Total daily physical activity
Physical activity (min)
Moderate-vigorous physical activity
Physical activity (min)
WHO
Female
Male
young
aged
