# Supplementary material for: Metabolic View on Human Healthspan: A Lipidome-Wide Association Study
Source: Metabolites. 2021 Apr 30;11(5):287. doi: 10.3390/metabo11050287 (PMC8146132; doi:10.3390/metabo11050287)
Supplement: Supplementary file 1 [file metabolites-11-00287-s001.zip › metabolites-1191027-supp-final/Fig_S3.pdf]

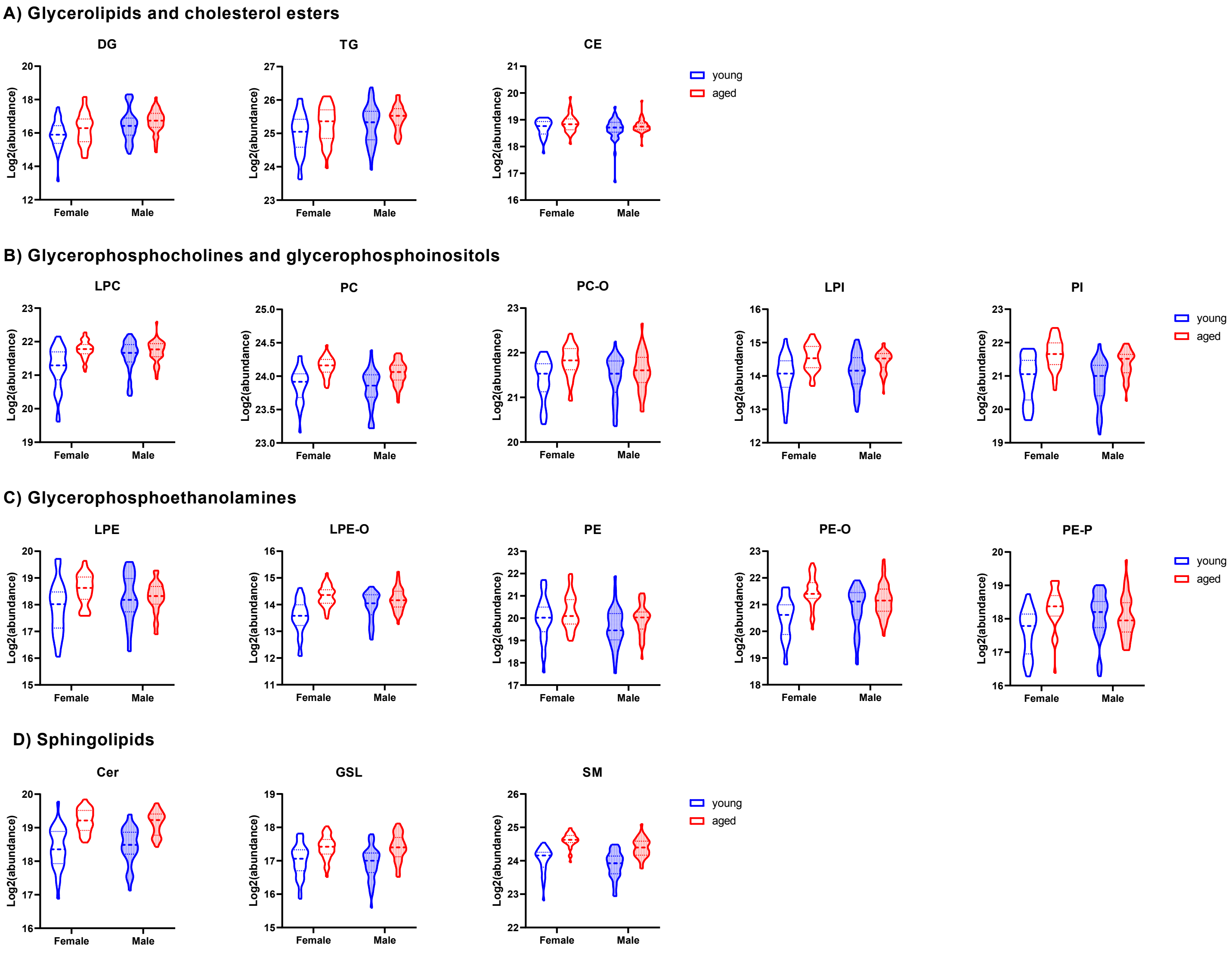
A) Glycerolipids and cholesterol esters
DG
TG
CE
young
aged
B) Glycerophosphocholines and glycerophosphoinositols
LPC
PC
PC-O
LPI
PI
young
aged
C) Glycerophosphoethanolamines
LPE
LPE-O
PE
PE-O
PE-P
young
aged
D) Sphingolipids
Cer
GSL
SM
young
aged
Log2(abundance)
Female
Male
