# Supplementary material for: Metabolic View on Human Healthspan: A Lipidome-Wide Association Study
Source: Metabolites. 2021 Apr 30;11(5):287. doi: 10.3390/metabo11050287 (PMC8146132; doi:10.3390/metabo11050287)
Supplement: Supplementary file 1 [file metabolites-11-00287-s001.zip › metabolites-1191027-supp-final/Supplementary fig and tables captions.pdf]

# Supplementary figures' and tables' captions

Figure S1 Distribution of clinical data by age and sex

Abbreviations: HbA1c = glycated haemoglobin, HDL = high-density lipoprotein, LDL = low-density lipoprotein, WHO = World Health Organization, indicates the WHO recommendations on daily physical activity time

Figure S2 Lipid subclasses: percentage of difference in the aged compared to the young

Abbreviations: BH = Benjamini-Hochberg, SM = sphingomyelins, PI = glycerophosphoinositols, GSL = glycosphingolipids, PC = glycerophosphocholines, Cer = ceramides, LPC = lyso-glycerophosphocholines, LPI = lyso-glycerophosphoinositols, LPE = lyso-glycerophosphoethanolamines, PC-O = alkyl-glycerophosphocholines, LPE-O = lyso-alkyl-glycerophosphoethanolamines, PE-O = alkyl-glycerophosphoethanolamines, PE = glycerophospoethanolamines, CE = cholesterol esters, PE-P = alkenyl-glycerophosphoethanolamines, DG = diglycerides and TG = triglycerides

Figure S3 Distribution of lipid subclasses by age and sex

Abbreviations: DG = diglycerides, TG = triglycerides, CE = cholesterol esters, LPC = lyso-glycerophosphocholines, PC = glycerophosphocholines, PC-O = alkyl-glycerophosphocholines, LPI = lyso-glycerophosphoinositols, PI = glycerophosphoinositols, LPE = lyso-glycerophosphoethanolamines, LPE-O = lyso-alkyl-glycerophosphoethanolamines, PE = glycerophospoethanolamines, PE-O = alkyl-glycerophosphoethanolamines, PE-P = alkenyl-glycerophosphoethanolamines, Cer = ceramides, GSL = glycosphingolipids, SM = sphingomyelins.

Figure S4 Associations between lipid saturation, age, sex and clinical variables

Abbreviations: BH = Benjamini-Hochberg, DG = diglycerides, TG = triglycerides, CE = cholesterol esters, LPC = lyso-glycerophosphocholines, PC = glycerophosphocholines, PC-O = alkyl-glycerophosphocholines, LPE = lyso-glycerophosphoethanolamines, LPE-O = lyso-alkyl-glycerophosphoethanolamines, PE = glycerophospoethanolamines, PE-O = alkyl-glycerophosphoethanolamines, PE-P = alkenyl-glycerophosphoethanolamines, LPI = lyso-glycerophosphoinositols, PI = glycerophosphoinositols, Cer = ceramides, GSL =

glycosphingolipids, SM = sphingomyelins, MUFA = monounsaturated fatty acids, PUFA = polyunsaturated fatty acids, HbA1c = glycated haemoglobin.

Figure S5 Saturation level: percentage of difference in the aged compared to the young

Abbreviations: BH = Benjamini-Hochberg, DG = diglycerides, TG = triglycerides, CE = cholesterol esters, LPC = lyso-glycerophosphocholines, PC = glycerophosphocholines, PC-O = alkyl-glycerophosphocholines, LPE = lyso-glycerophosphoethanolamines, LPE-O = lyso-alkyl-glycerophosphoethanolamines, PE = glycerophospoethanolamines, PE-O = alkyl-glycerophosphoethanolamines, PE-P = alkenyl-glycerophosphoethanolamines, LPI = lyso-glycerophosphoinositols, PI = glycerophosphoinositols, Cer = ceramides, GSL = glycosphingolipids, SM = sphingomyelins, MUFA = monounsaturated fatty acid, PUFA = polyunsaturated fatty acids.

Figure S6 Associations between PC/PE ratios, age, sex and clinical variables

Abbreviations: BH = Benjamini-Hochberg, LPC = lyso-glycerophosphocholines, LPE = lyso-glycerophosphoethanolamines, PC = glycerophosphocholines, PE = glycerophospoethanolamines, PC-O = alkyl-glycerophosphocholines, PE-O = alkyl-glycerophosphoethanolamines, HbA1c = glycated haemoglobin.

Figure S7 PC/PE ratios: percentage of difference in the aged compared to the young

Abbreviations: BH = Benjamini-Hochberg, LPC = lyso-glycerophosphocholines, LPE = lyso-glycerophosphoethanolamines, PC = glycerophosphocholines, PE = glycerophospoethanolamines, PC-O = alkyl-glycerophosphocholines, PE-O = alkyl-glycerophosphoethanolamines.

Figure S8 Lipid subclasses: percentage of difference in females compared to males

Abbreviations: BH = Benjamini-Hochberg, SM = sphingomyelins, PI = glycerophosphoinositols, GSL = glycosphingolipids, PC = glycerophosphocholines, Cer = ceramides, LPC = lyso-glycerophosphocholines, LPI = lyso-glycerophosphoinositols, LPE = lyso-glycerophosphoethanolamines, PC-O = alkyl-glycerophosphocholines, LPE-O = lyso-alkyl-glycerophosphoethanolamines, PE-O = alkyl-glycerophosphoethanolamines, PE =

glycerophospoethanolamines, CE = cholesterol esters, PE-P = alkenyl-glycerophosphoethanolamines, DG = diglycerides and TG = triglycerides

Figure S9 Lipid species: percentage of difference in females compared to males

A) Young

B) Aged

Abbreviations: BH = Benjamini-Hochberg, CE = cholesterol ester, Cer = ceramide, GSL = glycosphingolipids, LPC = lyso-glycerophosphocholines, LPE = lyso-glycerophosphoethanolamines, LPE-O = lyso-alkyl-glycerophosphoethanolamines, LPI = lyso-glycerophosphoinositols, PI = glycerophosphoinositols, PC = glycerophosphocholines, PC-O = alkyl-glycerophosphocholines, PE = glycerophospoethanolamines, PE-O = alkyl-glycerophosphoethanolamines, PI = glycerophosphoinositols, SM = sphingomyelins and TG = triglycerides.

Figure S10 Saturation level: percentage of difference in females compared to males

Abbreviations: BH = Benjamini-Hochberg, DG = diglycerides, TG = triglycerides, CE = cholesterol esters, LPC = lyso-glycerophosphocholines, PC = glycerophosphocholines, PC-O = alkyl-glycerophosphocholines, LPE = lyso-glycerophosphoethanolamines, LPE-O = lyso-alkyl-glycerophosphoethanolamines, PE = glycerophospoethanolamines, PE-O = alkyl-glycerophosphoethanolamines, PE-P = alkenyl-glycerophosphoethanolamines, LPI = lyso-glycerophosphoinositols, PI = glycerophosphoinositols, Cer = ceramides, GSL = glycosphingolipids, SM = sphingomyelins, MUFA = monounsaturated fatty acid, PUFA = polyunsaturated fatty acids.

Figure S11 PC/PE ratios: percentage of difference in females compared to males

Abbreviations: BH = Benjamini-Hochberg, LPC = lyso-glycerophosphocholines, LPE = lyso-glycerophosphoethanolamines, PC = glycerophosphocholines, PE = glycerophospoethanolamines, PC-O = alkyl-glycerophosphocholines, PE-O = alkyl-glycerophosphoethanolamines.

Figure S12 LION enrichment analysis: females compared to males

A) Young

B) Aged

Abbreviations: LION = Lipid Ontology, FDR = false discovery rate

Table S1 Principal component analysis highlighting the difference in lipid profile between young and aged participants

Abbreviations: PC1 = principal component 1, PC2 = principal component 2

Table S2 Differential analysis highlighting lipid species displaying significant higher levels in the aged compared to the young participants

Abbreviations: BH = Benjamini-Hochberg

Table S3 Multiple linear regressions on the subclass level

Abbreviations: BH = Benjamini-Hochberg

Table S4 Multiple linear regressions on the species level

Abbreviations: BH = Benjamini-Hochberg

Table S5 Post-hoc tests on the subclass level

Abbreviations: mean_age_diff_f = mean age difference within females, mean_age_diff_m = mean age difference within males, mean_sex_diff_y = mean sex difference within young, mean_sex_diff_a = mean sex difference within aged, p_mean_diff_age_f = p-value mean age difference within females, p_mean_diff_age_m = p-value mean age difference within males, p_mean_diff_sex_y = p-value mean sex difference within young, p_mean_diff_sex_a = p-value mean sex difference within aged, p_val_age_f_adj = adjusted p-value mean age difference within females, p_val_age_m_adj = adjusted p-value mean age difference within males, p_val_sex_y_adj = adjusted p-value mean sex difference within young, p_val_sex_a_adj = adjusted p-value mean sex difference within aged, p_val_age_f_adj_cat = categoric adjusted p-

value mean age difference within females, p_val_age_m_adj_cat = categoric adjusted p-value mean age difference within males, p_val_sex_y_adj_cat = categoric adjusted p-value mean sex difference within young, p_val_sex_a_adj_cat = categoric adjusted p-value mean sex difference within aged

Table S6 Post-hoc tests on the species level

Abbreviations: mean_age_diff_f = mean age difference within females, mean_age_diff_m = mean age difference within males, mean_sex_diff_y = mean sex difference within young, mean_sex_diff_a = mean sex difference within aged, p_mean_diff_age_f = p-value mean age difference within females, p_mean_diff_age_m = p-value mean age difference within males, p_mean_diff_sex_y = p-value mean sex difference within young, p_mean_diff_sex_a = p-value mean sex difference within aged, p_val_age_f_adj = adjusted p-value mean age difference within females, p_val_age_m_adj = adjusted p-value mean age difference within males, p_val_sex_y_adj = adjusted p-value mean sex difference within young, p_val_sex_a_adj = adjusted p-value mean sex difference within aged, p_val_age_f_adj_cat = categoric adjusted p-value mean age difference within females, p_val_age_m_adj_cat = categoric adjusted p-value mean age difference within males, p_val_sex_y_adj_cat = categoric adjusted p-value mean sex difference within young, p_val_sex_a_adj_cat = categoric adjusted p-value mean sex difference within aged

Table S7 Multiple linear regressions regarding the MUFA/PUFA ratios

Abbreviations: BH = Benjamini-Hochberg

Table S8 Post-hoc tests regarding the MUFA/PUFA ratios

Abbreviations: mean_age_diff_f = mean age difference within females, mean_age_diff_m = mean age difference within males, mean_sex_diff_y = mean sex difference within young, mean_sex_diff_a = mean sex difference within aged, p_mean_diff_age_f = p-value mean age difference within females, p_mean_diff_age_m = p-value mean age difference within males, p_mean_diff_sex_y = p-value mean sex difference within young, p_mean_diff_sex_a = p-value mean sex difference within aged, p_val_age_f_adj = adjusted p-value mean age difference within females, p_val_age_m_adj = adjusted p-value mean age difference within males, p_val_sex_y_adj = adjusted p-value mean sex difference within young, p_val_sex_a_adj = adjusted p-value mean sex difference within aged, p_val_age_f_adj_cat = categoric adjusted p-

value mean age difference within females, p_val_age_m_adj_cat = categoric adjusted p-value mean age difference within males, p_val_sex_y_adj_cat = categoric adjusted p-value mean sex difference within young, p_val_sex_a_adj_cat = categoric adjusted p-value mean sex difference within aged

Table S9 Multiple linear regressions regarding the PC/PE ratios

Abbreviations: BH = Benjamini-Hochberg

Table S10 Post-hoc tests regarding the PC/PE ratios

Abbreviations: mean_age_diff_f = mean age difference within females, mean_age_diff_m = mean age difference within males, mean_sex_diff_y = mean sex difference within young, mean_sex_diff_a = mean sex difference within aged, p_mean_diff_age_f = p-value mean age difference within females, p_mean_diff_age_m = p-value mean age difference within males, p_mean_diff_sex_y = p-value mean sex difference within young, p_mean_diff_sex_a = p-value mean sex difference within aged, p_val_age_f_adj = adjusted p-value mean age difference within females, p_val_age_m_adj = adjusted p-value mean age difference within males, p_val_sex_y_adj = adjusted p-value mean sex difference within young, p_val_sex_a_adj = adjusted p-value mean sex difference within aged, p_val_age_f_adj_cat = categoric adjusted p-value mean age difference within females, p_val_age_m_adj_cat = categoric adjusted p-value mean age difference within males, p_val_sex_y_adj_cat = categoric adjusted p-value mean sex difference within young, p_val_sex_a_adj_cat = categoric adjusted p-value mean sex difference within aged

Table S11 LION enrichment analysis, age difference within females

Abbreviations: LION = lipid ontology, FDR q-value = false discovery rate q-value

Table S12 LION enrichment analysis, age difference within males

Abbreviations: LION = lipid ontology, FDR q-value = false discovery rate q-value

Table S13 LION enrichment analysis, sex difference within young

Abbreviations: LION = lipid ontology, FDR q-value = false discovery rate q-value

<u>Table S14 LION enrichment analysis, sex difference within aged</u>

Abbreviations: LION = lipid ontology, FDR q-value = false discovery rate q-value

<u>Table S15 Lipidomics and clinical dataset</u>

Abbreviations: PA = physical activity, HbA1c = glycated haemoglobin, Chol = total cholesterol, TAG = triglycerides, LDL-C = low density lipoprotein cholesterol, HDL-C = low density lipoprotein cholesterol, NSAID = nonsteroidal anti-inflammatory drug, Z-drugs = nonbenzodiazepine benzodiazepine receptor agonists.
