# Supplementary figures and images for: Metabolic View on Human Healthspan: A Lipidome-Wide Association Study
Source: Metabolites. 2021 Apr 30;11(5):287. doi: 10.3390/metabo11050287 (PMC8146132; doi:10.3390/metabo11050287)

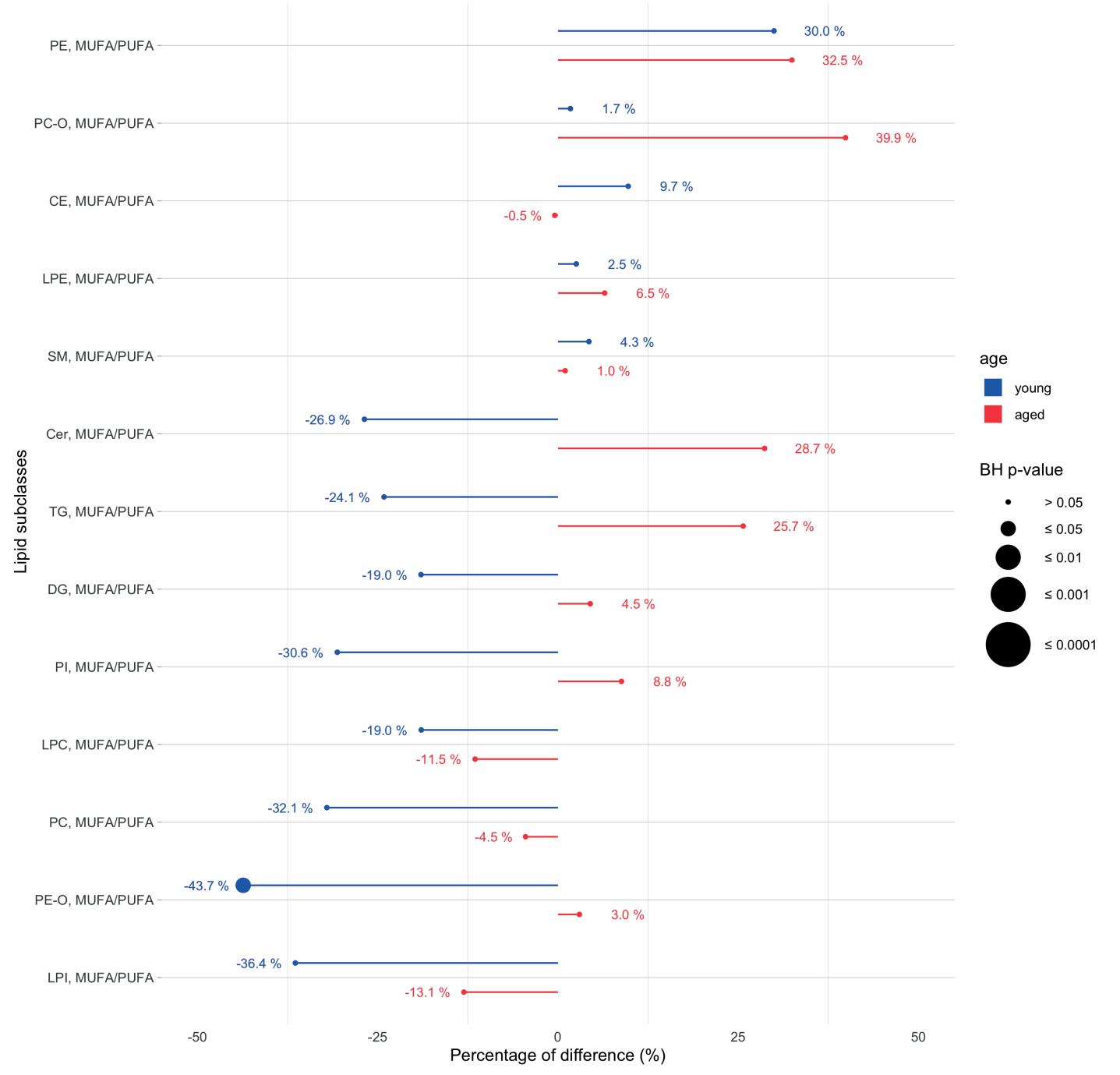

Supplement: Supplementary file 1 [file metabolites-11-00287-s001.zip › metabolites-1191027-supp-final/Fig_S10.pdf]

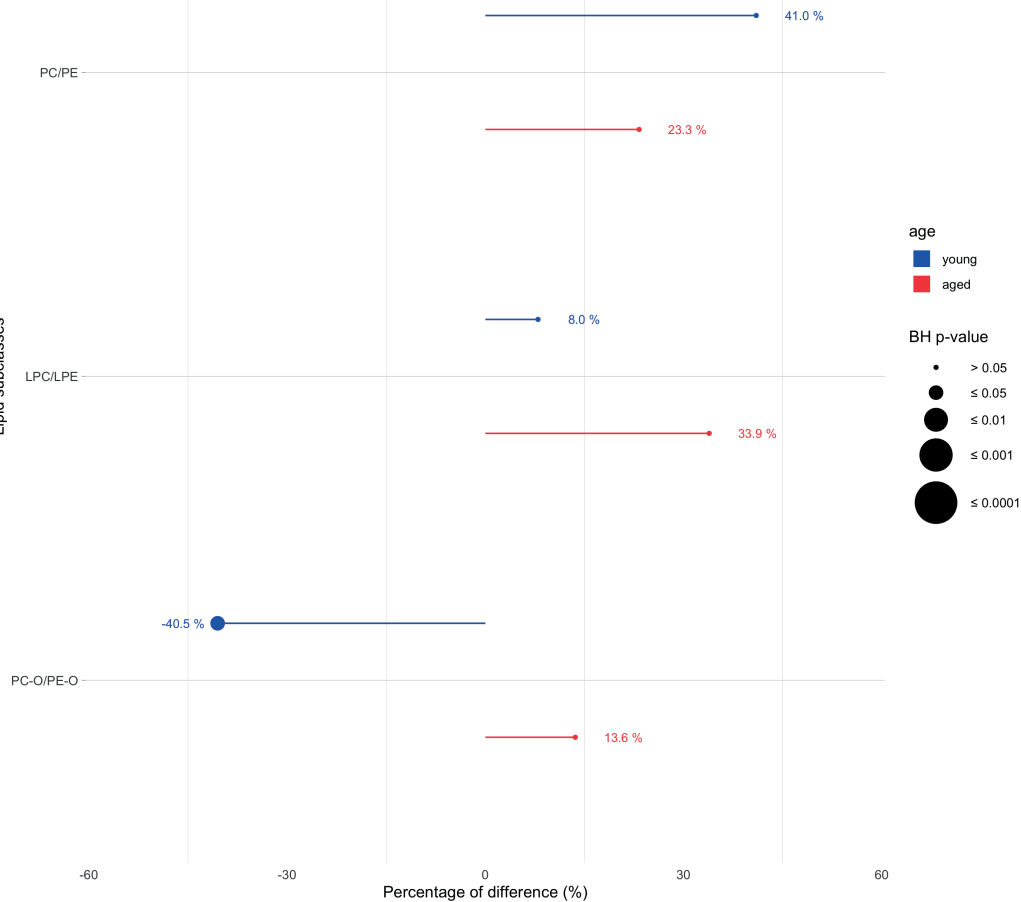

Supplement: Supplementary file 1 [file metabolites-11-00287-s001.zip › metabolites-1191027-supp-final/Fig_S11.pdf]

# A Young

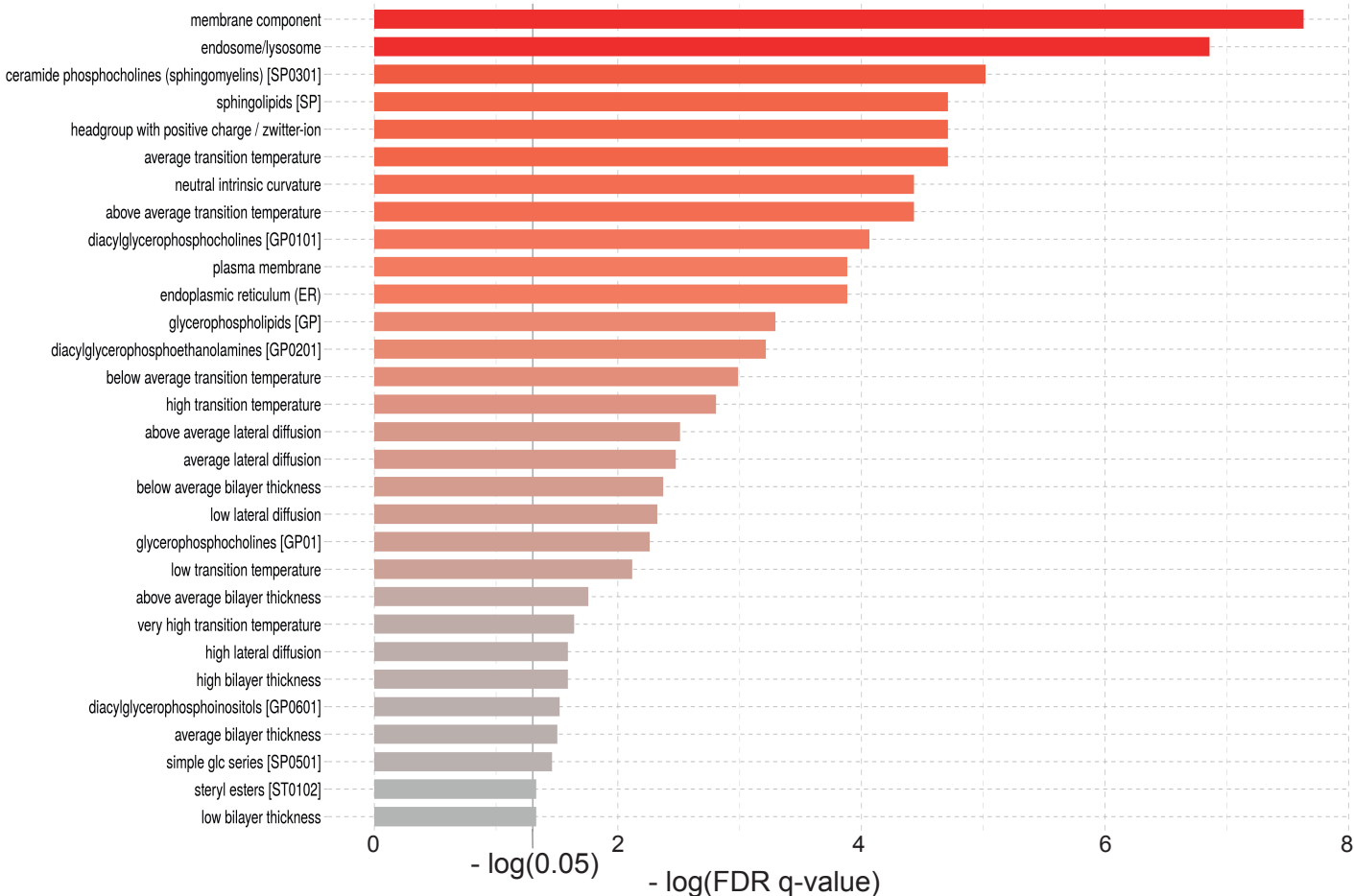

# B Aged

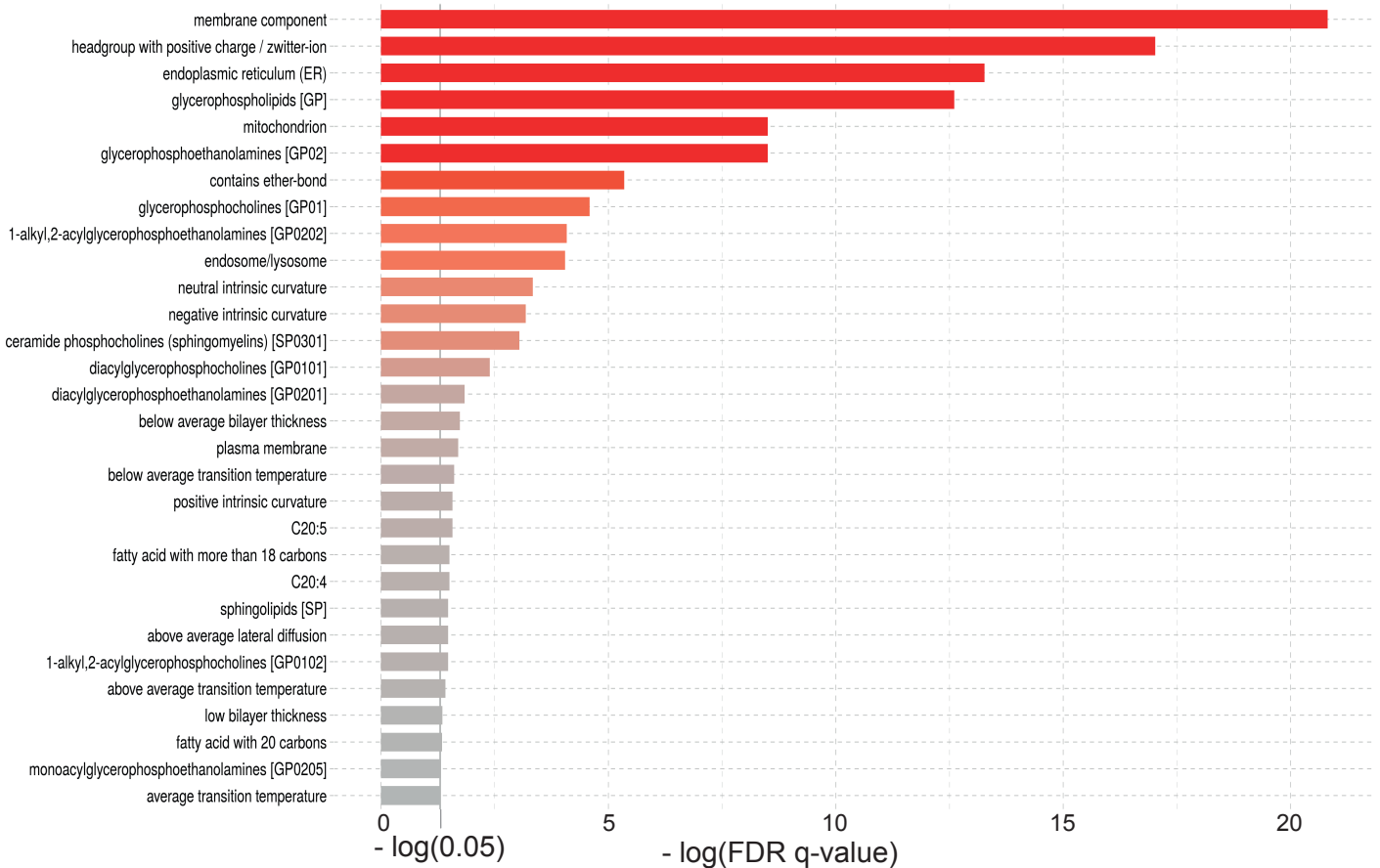

Supplement: Supplementary file 1 [file metabolites-11-00287-s001.zip › metabolites-1191027-supp-final/Fig_S12.pdf]

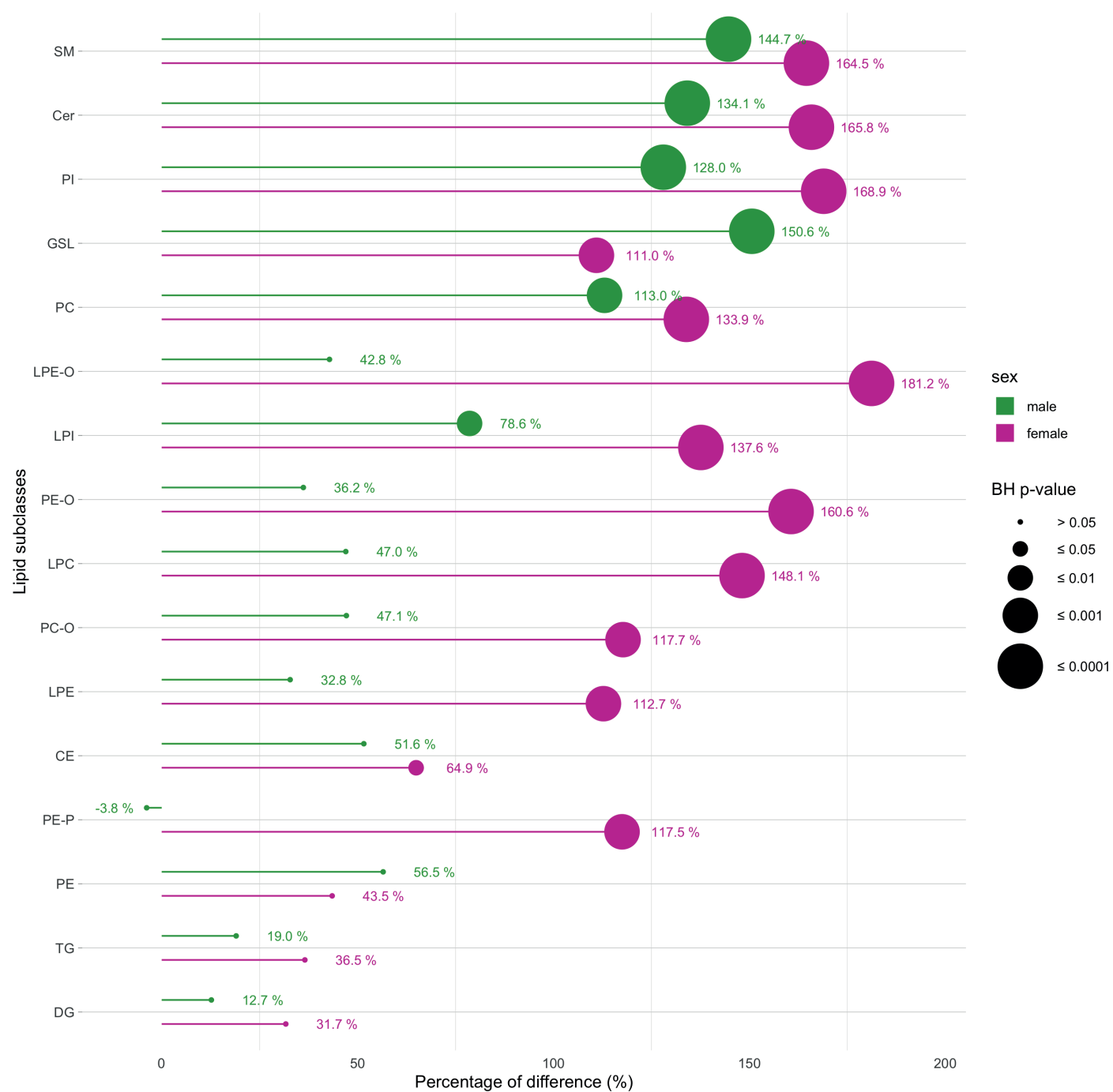

Supplement: Supplementary file 1 [file metabolites-11-00287-s001.zip › metabolites-1191027-supp-final/Fig_S2.pdf]

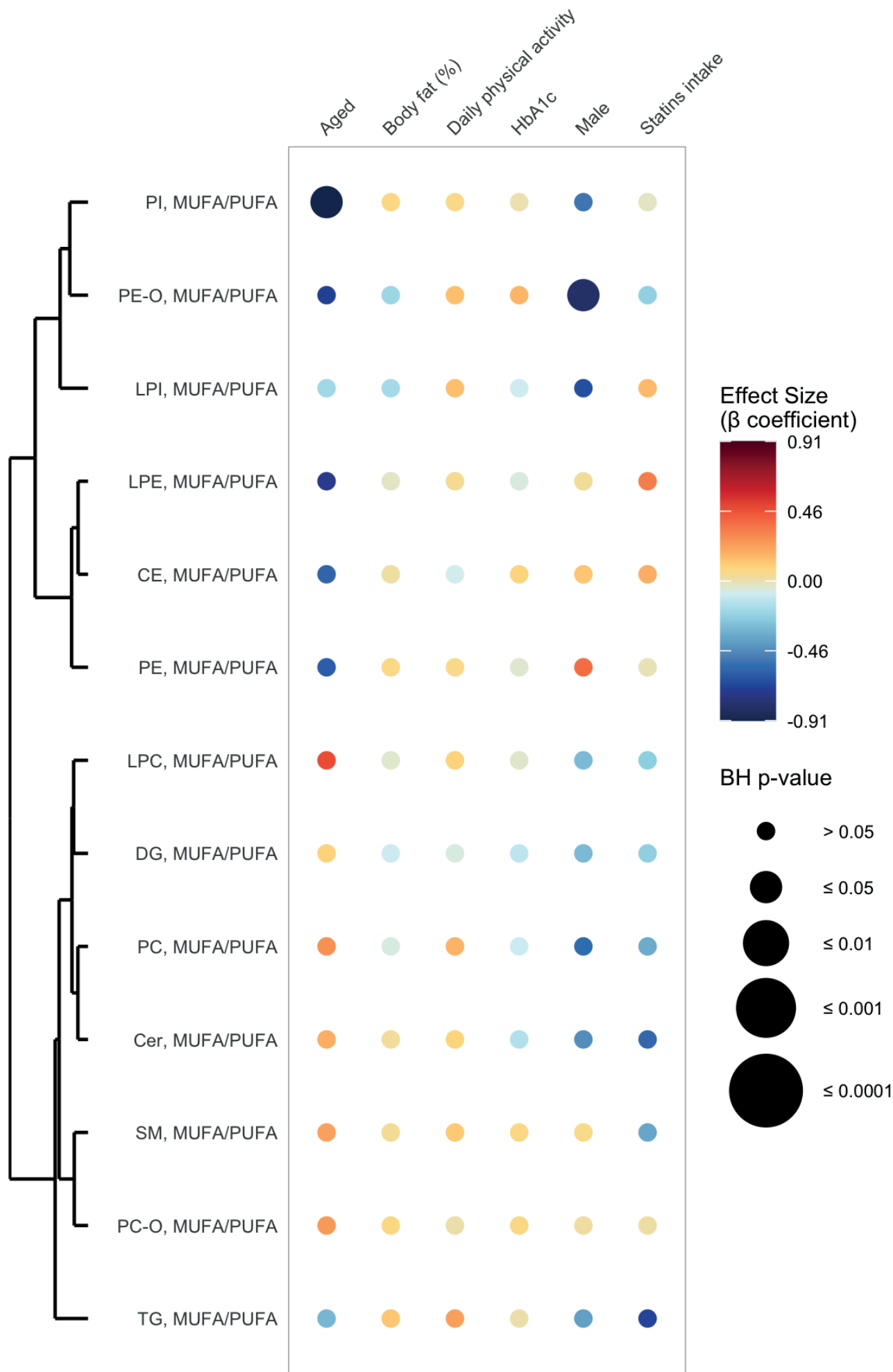

Supplement: Supplementary file 1 [file metabolites-11-00287-s001.zip › metabolites-1191027-supp-final/Fig_S4.pdf]

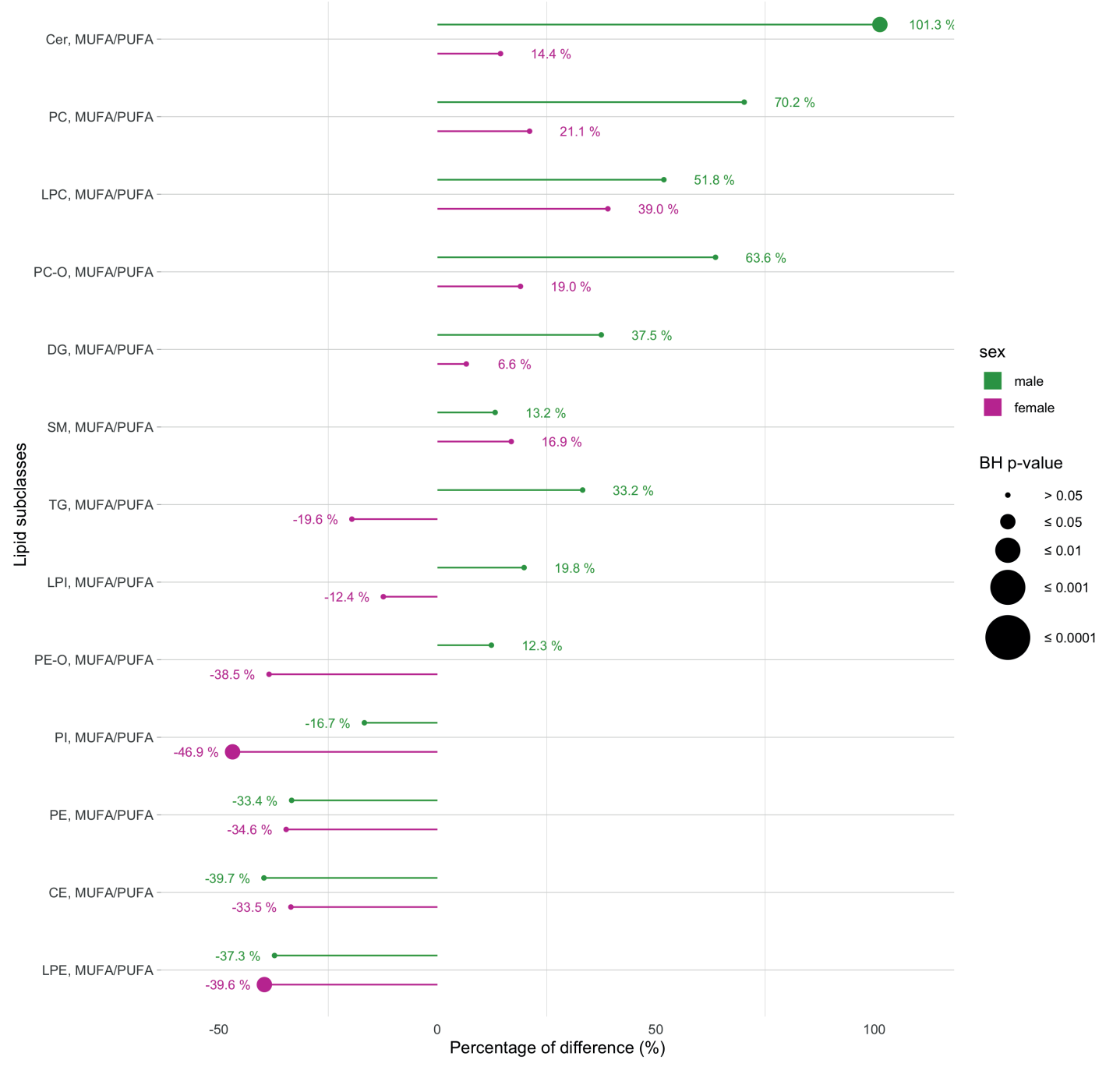

Supplement: Supplementary file 1 [file metabolites-11-00287-s001.zip › metabolites-1191027-supp-final/Fig_S5.pdf]

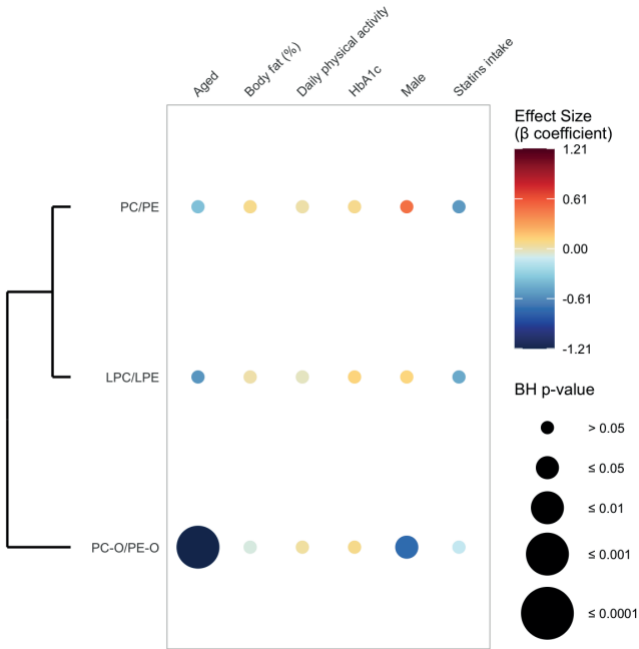

Supplement: Supplementary file 1 [file metabolites-11-00287-s001.zip › metabolites-1191027-supp-final/Fig_S6.pdf]

Lipid subclasses

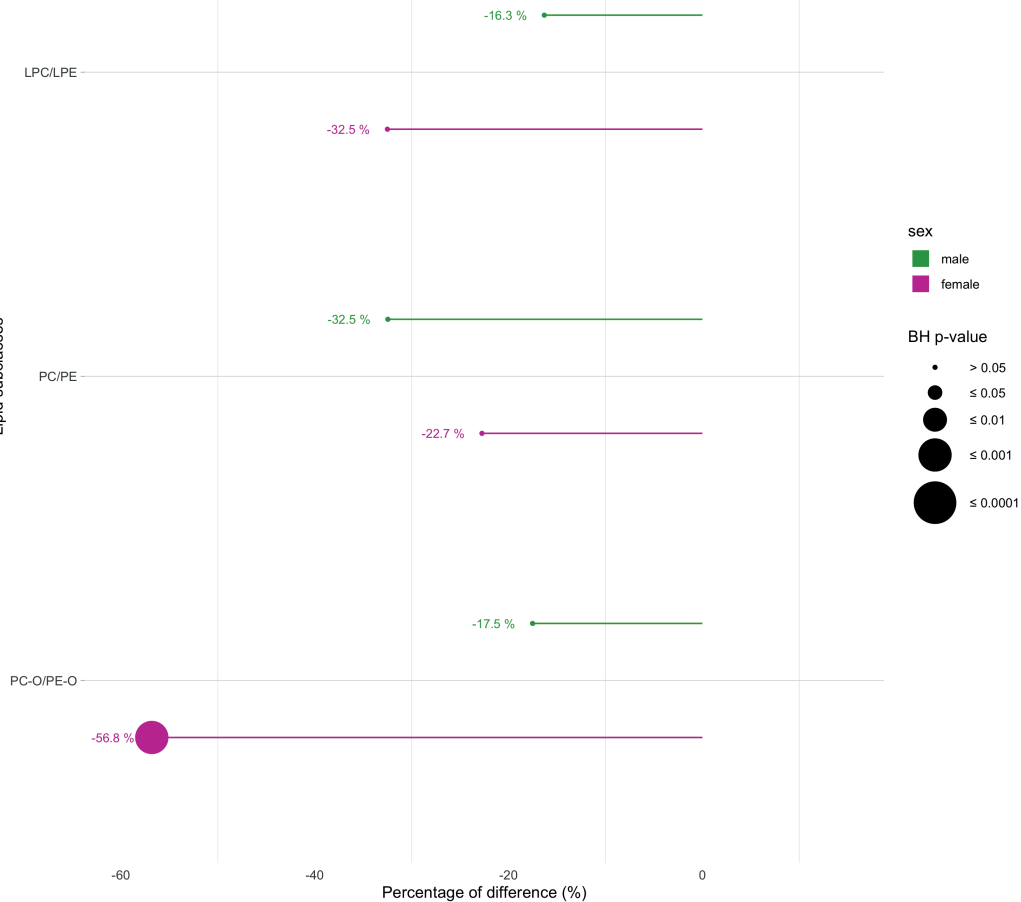

Supplement: Supplementary file 1 [file metabolites-11-00287-s001.zip › metabolites-1191027-supp-final/Fig_S7.pdf]

**A**

## Young

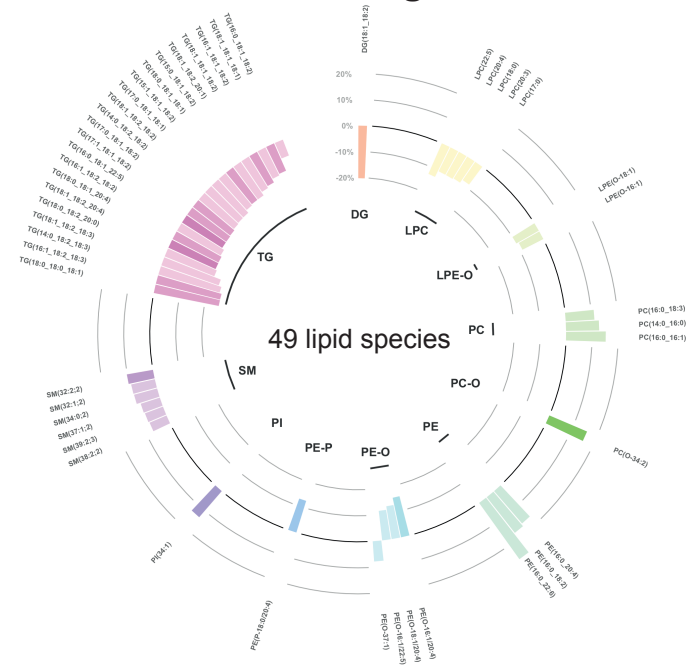

# B

## Aged

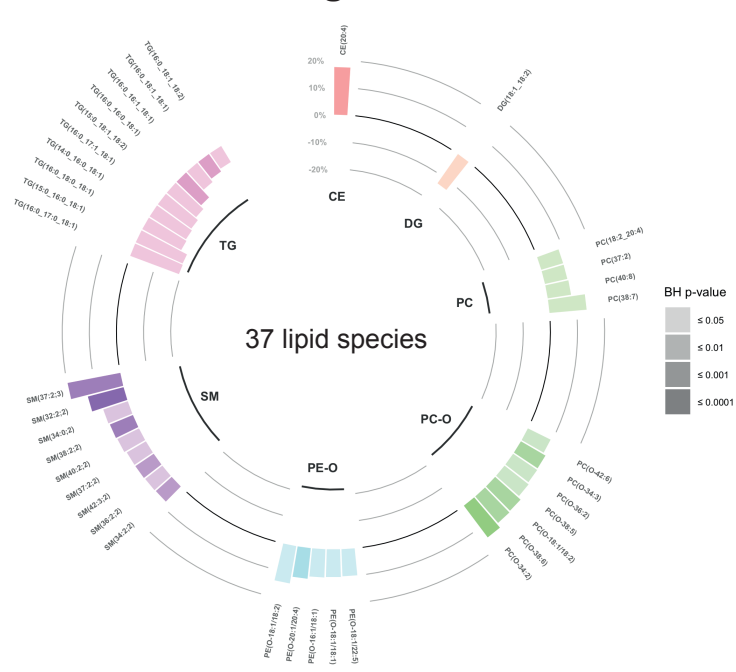

Supplement: Supplementary file 1 [file metabolites-11-00287-s001.zip › metabolites-1191027-supp-final/Fig_S8.pdf]

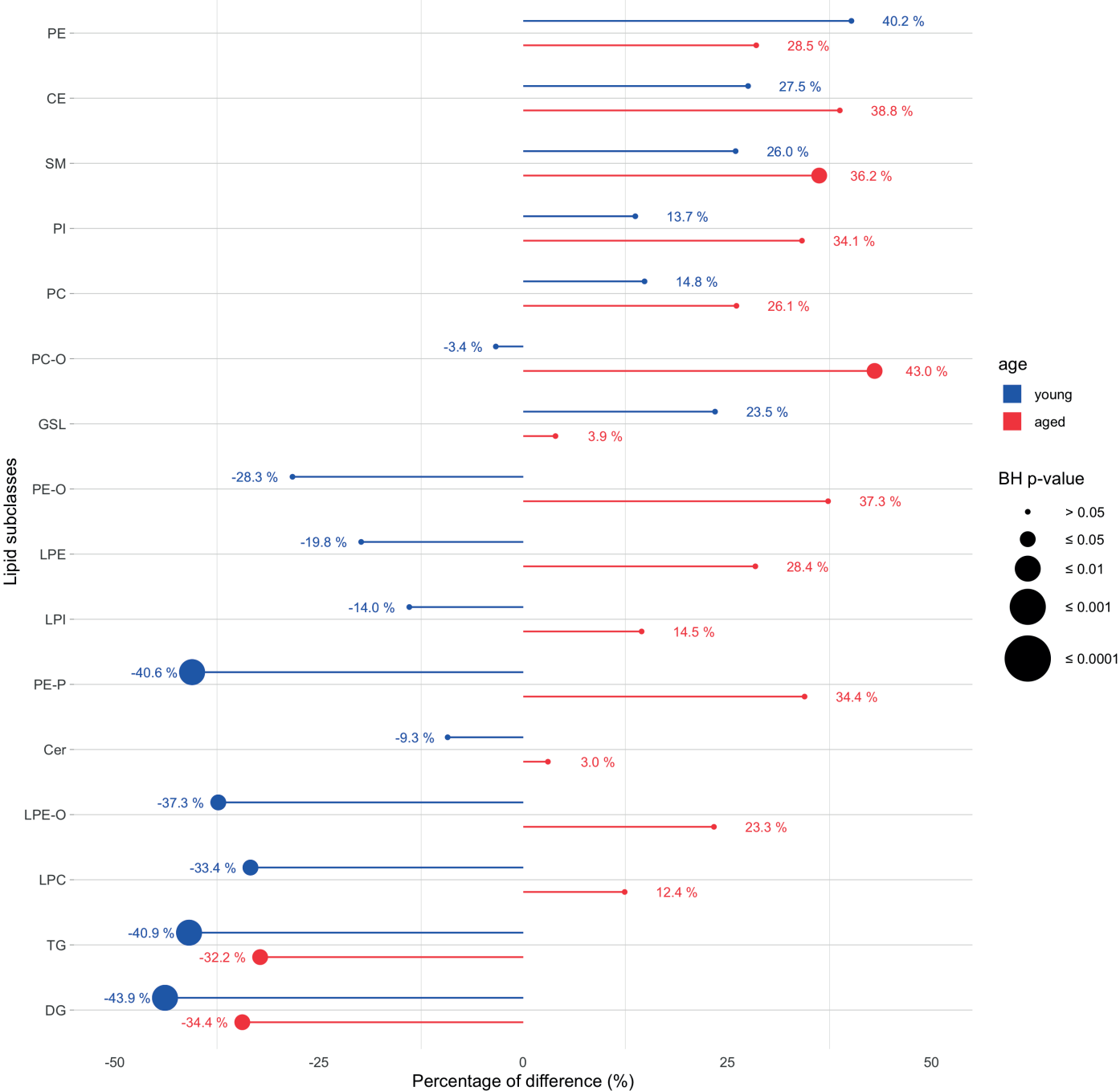

Supplement: Supplementary file 1 [file metabolites-11-00287-s001.zip › metabolites-1191027-supp-final/Fig_S9.pdf]
